# Complete abdominal aortic aneurysm thrombosis and obstruction of both common iliac arteries with intrathrombotic pressures demonstrating a continuing risk of rupture: a case report and review of the literature

**DOI:** 10.1186/1752-1947-3-9292

**Published:** 2009-11-24

**Authors:** Konstantinos A Filis, Emmanuel E Lagoudianakis, Haridimos Markogiannakis, Aikaterini Kotzadimitriou, Nikolaos Koronakis, Konstantinos Bramis, Konstantinos Xiromeritis, Dimitrios Theodorou, Andreas Manouras

**Affiliations:** 1 1st Department of Propaedeutic Surgery, Hippokrateion Hospital, Athens Medical School, University of Athens, Athens, Greece; 2 Second Department of Surgery, 417 NIMTS (Military Veterans' Fund Hospital), Athens, Greece

## Abstract

**Introduction:**

Although mural thrombus in an abdominal aortic aneurysm is frequent and its role has been studied extensively, complete thrombosis of an abdominal aneurysm is extremely rare and its natural history in relation to the risk of rupture is not known. The case of a patient with a completely thrombosed infrarenal aneurysm is presented along with a literature review.

**Case presentation:**

We report the case of a 56-year-old Caucasian man with an infrarenal abdominal aortic aneurysm, presenting at our hospital due to critical ischemia of his right lower limb. Computed tomography and angiography demonstrated complete aneurysm thrombosis and obstruction of both common iliac arteries.

**Conclusion:**

During the operation, systolic and mean intrathrombotic pressures, measured in different levels, constituted 74.5-90.2% and 77.5-92.5% of systolic and mean intraluminal pressure and 73-88.4% and 76.5-91.3% of systemic pressure, respectively. Our findings show that there may be a continuing risk of rupture in cases of a thrombosed abdominal aortic aneurysm.

## Introduction

Although thrombus formation is common within an abdominal aortic aneurysm, there is still controversy over the role that aneurysmal mural thrombus plays in the risk of rupture. Complete thrombosis of an aneurysm is extremely rare and its role in abdominal aortic aneurysm rupture is still not clear. We report our findings regarding intrathrombotic pressures of a completely thrombosed infrarenal aneurysm and their implications for the risk of rupture.

## Case presentation

**Figure 1:**
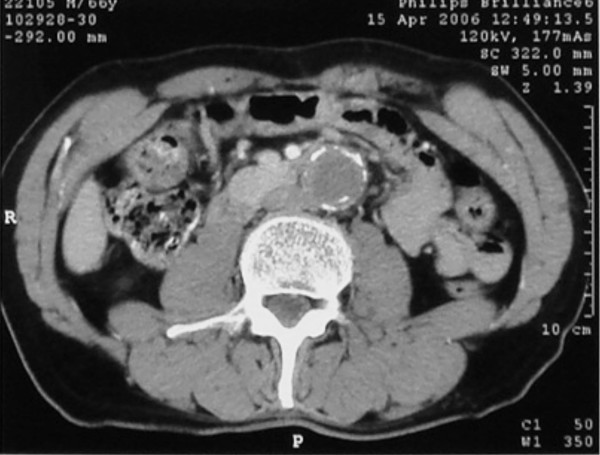
**Contrast-enhanced computed tomography scan revealing complete thrombosis of the aneurysm extending from the infrarenal aortic neck to both common, external and internal iliac arteries**. Maximal aneurysm diameter was 4.3 cm.

**Figure 2:**
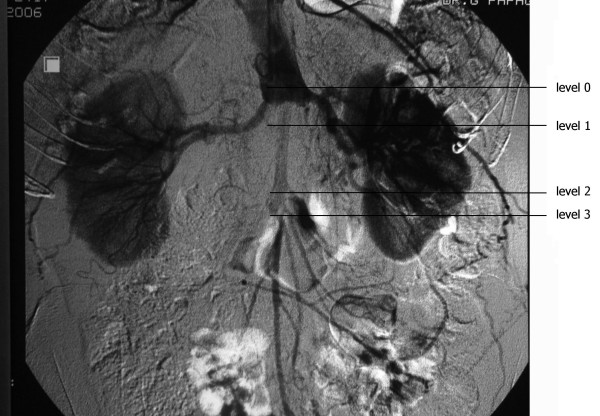
**Digital subtraction angiography demonstrating complete aneurysm thrombosis and obstruction of both common iliac arteries**. Level 0: 1 cm above the renal arteries; level 1: 3 cm distal to the renal arteries; level 2: at the origin of the inferior mesentery artery; level 3: 2 cm distal to level 2.

A 56-year-old Caucasian man with a known 5-year history of an infrarenal abdominal aortic aneurysm was admitted due to critical ischemia of his right lower limb. The patient's left leg had been amputated at the left common femoral artery level (very high femoral amputation) following a car accident 25 years previously. Abdominal computed tomography (CT) 8 months earlier showed a 4 cm infrarenal aneurysm and severe atherosclerosis of both common iliac arteries. Physical examination on admission revealed absent femoral pulses bilaterally with an ankle-brachial index (ABI) on the right of 0.50. A contrast-enhanced CT scan demonstrated complete aneurysm thrombosis extending from the aortic neck to both common, external and internal iliac arteries. Maximal aneurysm diameter was 4.3 cm (Figure 1). Digital subtraction angiography (DSA) confirmed complete aneurysm thrombosis and obstruction of both common iliac arteries (Figure 2).

**Figure 3:**
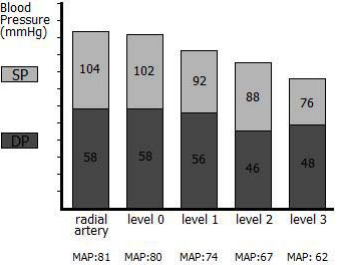
**Systolic blood pressure (SP) and diastolic blood pressure (DP) on the vertical axis at different levels**. Level 0: 1 cm above the renal arteries; level 1: 3 cm distal to the renal arteries; level 2: at the origin of the inferior mesentery artery; level 3: 2 cm distal to level 2. Mean arterial blood pressure (MAP) is shown on the horizontal axis.

Intra-operatively, after dissection of the aneurysm and before aortic clamping, intrathrombotic pressures were simultaneously measured at three levels, with 18-gauge needles inserted into the thrombus 3 cm distal to the renal arteries (level 1), at the origin of the inferior mesenteric artery (level 2), and 2 cm distal to level 2 (level 3). Systemic arterial blood pressure and intraluminal pressure were also simultaneously measured by a radial catheter and an 18-gauge needle, respectively, 1 cm above the renal arteries (level 0) (Figure 2). Table 1 shows systemic, intraluminal, and intrathrombotic pressures and systolic and mean intrathrombotic pressures at the three levels as percentages of the systolic and mean intraluminal and systemic pressures (also Figure 3).

**Table 1:** Intraluminal and intrathrombotic pressures of the abdominal aortic aneurysm

Measurement	Radial artery	Level 0	Level 1	Level 2	Level 3
Systolic (mmHg)	104	102	92	88	76
Percentage of radial artery (%)			88.4%	84.6%	73%
Percentage of level 0 (%)			90.2%	86.2%	74.5%
Diastolic (mmHg)	58	58	56	46	48
Mean (mmHg)	81	80	74	67	62
Percentage of radial artery (%)			91.3%	82.7%	76.5%
Percentage of level 0 (%)			92.5%	83.7%	77.5%

Level 0: 1 cm above the renal arteries; level 1: 3 cm distal to the renal arteries; level 2: at the origin of the inferior mesentery artery; level 3: 2 cm distal to level 2.

After the completion of measurements, the aneurysm was repaired by standard aortofemoral grafting. Aortobifemoral bypass is the procedure of choice for the majority of patients with aorto-iliac occlusive disease. In our patient, an aortofemoral bypass was used to treat the right-limb-threatening ischemia secondary to atherosclerotic occlusive disease of both common, internal and external iliac arteries and the completely thrombosed aneurysm extending from the infrarenal aorta to both iliac systems. The patient was anesthetized with a combined general and epidural technique. Dissection was carried out directly onto the anterior surface of the common femoral artery and continued cephalad to the inguinal ligament and caudally to expose the common femoral bifurcation and proximal ends of both superficial and profunda femoral arteries. The infrarenal aorta was exposed by a midline vertical incision, with retraction of the abdominal organs and incision of the posterior parietal peritoneum. After dissection, retroperitoneal tunnels were made for the passage of each graft limb from the aorta to the groin. After applying proximal and distal vascular clamps, the aorta was incised longitudinally to remove the thrombus and then incised horizontally to perform an end-to-end proximal anastomosis of the infrarenal aorta (under the renal vein) to a polytetrafluoroethylene (PTFE) bifurcated prosthetic graft. The distal end-to-side anastomosis was performed placing the toe of the graft on the profunda femoral artery beyond the diseased femoral artery.

The patient had the surgery two days after admission, had an uneventful recovery and was discharged on the seventh postoperative day.

## Discussion

Abdominal aortic aneurysms rupture when the aortic wall stress exceeds the strength of the vascular tissue. Stress in the vessel wall depends on blood pressure, the diameter of the vessel, and the thickness of the wall; therefore, intrathrombotic pressure is of great importance. In our case, systolic and mean intrathrombotic pressures constituted 74.5-90.2% and 77.5-92.5% of the systolic and mean intraluminal pressure and 73-88.4% and 76.5-91.3% of the systemic pressure, respectively. Our results show that there may be a continuing risk of rupture in cases of a thrombosed abdominal aortic aneurysm.

The role of intraluminal thrombus of an aneurysm in relation to the risk of rupture is controversial. Reports describing reduction and increase in rupture risk can be found in the literature. Intrathrombotic pressure approximated intraluminal pressure in excluded abdominal aortic aneurysms after proximal aortic neck and distal iliac clamping [1], while Schurink *et al. *found that an aneurysm thrombus did not reduce either the mean or pulse pressure near the aneurysmal wall [2]. Both studies concluded that a thrombus within the aneurysm may not reduce the risk of its rupture [1,2]. In a completely thrombosed abdominal aortic aneurysm, the percentages of the systolic and mean intrathrombotic pressures to the intraluminal pressure were 88-90% and 95-99%, respectively. These results imply that a mural aneurysmal thrombus does not significantly decrease the pressure on the aneurysmal wall, even in a thrombosed aneurysm. Our findings are in agreement with those of Schurink, Takagi and colleagues [2,3].

However, several studies support the view that the thrombus may prevent the aneurysm from rupture by reducing the aneurysmal wall stress [4,5]. In contrast, it has also been suggested that the presence of a thrombus may increase the risk of rupture. In particular, the thickness of the thrombus has been associated with the risk of rupture [6], while growth of the thrombus has been associated with a higher likelihood of rapid abdominal aortic aneurysm expansion [7] and rupture [8]. Our patient had a thrombus of approximately 4 cm in maximum diameter that exhibited continuous transition, consisting of multiple layers forming a single structure. The layers of this thrombus were firmly attached to each other and the transition of the luminal to the medial region was spread over several layers. The transition from medial to abluminal regions was also continuous.

Moreover, an intraluminal thrombus has been shown to alter the normal pattern of oxygen supply to the aneurysm wall leading to local hypoxia, which may further lead to wall weakening and increased potential for rupture [9]. Compared with the thrombus free wall, the aneurysm wall covered by the thrombus is thinner, and contains fewer and fragmented elastin fibers as well as fewer, and more apoptotic or necrotic, smooth muscle cells, but more inflammatory cells. This implies that the thrombus may affect the structural integrity and stability of the wall by influencing the degradation of the extracellular matrix of the underlying vessel wall and thereby predisposing it for rupture [10].

## Conclusion

The role of complete thrombosis in abdominal aortic aneurysm rupture is still not clear. It has been documented, however, that a thrombosed abdominal aortic aneurysm can rupture [11-15]. The first reported case of late rupture of such an aneurysm was described in 1984 [11], the patient had a spontaneously thrombosed aneurysm and died of rupture 5 1/2 months after diagnosis of thrombosis. Rupture of a thrombosed abdominal aneurysm 2 years following diagnosis [12], as well as of a thoracoabdominal aneurysm 7 months after treatment with an axillobifemoral bypass [13], have also been reported. Additionally, late rupture of an abdominal aortic aneurysm that had been successfully treated by surgically induced thrombosis has been described [14,15]. These cases may support the thesis that complete thrombosis of an aneurysm does not preclude risk of rupture.

In conclusion, our findings regarding intrathrombotic pressure show that a complete thrombosis of an abdominal aortic aneurysm does not significantly decrease the pressure on the aneurysmal wall. Therefore, thrombosed aneurysms may remain at risk of rupture and need close monitoring as well as appropriate management.

## Abbreviations

ABI: ankle-brachial index; CT: computed tomography; DSA: digital subtraction angiography; PTFE: polytetrafluoroethylene

## Consent

Written informed consent was obtained from the patient for publication of this case report and any accompanying images. A copy of the written consent is available for review by the Editor-in-Chief of this journal.

## Competing interests

The authors declare that they have no competing interests.

## Authors' contributions

KAF, DT and AM contributed to revision and drafting of the manuscript. EEL, HM, NK and KX contributed to data collection, interpretation of data and the literature search. AK and KB contributed to the literature search and drafting of the manuscript. All authors read and approved the final manuscript.
